# Metabolic engineering of *Escherichia coli* to optimize melanin synthesis from glucose

**DOI:** 10.1186/1475-2859-12-108

**Published:** 2013-11-13

**Authors:** María I Chávez-Béjar, Victor E Balderas-Hernandez, Aída Gutiérrez-Alejandre, Alfredo Martinez, Francisco Bolívar, Guillermo Gosset

**Affiliations:** 1 Departamento de Ingeniería Celular y Biocatálisis, Instituto de Biotecnología, Universidad Nacional Autónoma de México, Apdo. Postal 510-3, Cuernavaca, Morelos CP 62271, México; 2 Laboratorio de Biología Integrativa de Plantas y Microorganismos, Unidad Académica de Ciencias Biológicas, Universidad Autónoma de Zacatecas, Av. Preparatoria s/n, Col. Agronómica, CP 98066, Zacatecas, México; 3 UNICAT, Departamento de Ingeniería Química, Facultad de Química, Universidad Nacional Autónoma de México, México, DF, México

**Keywords:** Melanin, L-tyrosine, Tyrosinase, Metabolic engineering, *Escherichia coli*

## Abstract

**Background:**

Natural aromatic polymers, mainly melanins, have potential and current applications in the cosmetic, pharmaceutical and chemical industries. The biotechnological production of this class of compounds is based on tyrosinase-dependent conversion of L-tyrosine and other aromatic substrates into melanins. The purpose of this work was to apply metabolic engineering for generating *Escherichia coli* strains with the capacity to synthesize an aromatic polymer from a simple carbon source.

**Results:**

The strategy was based on the expression in *E. coli* of the Mut*melA* gene from *Rhizobium etli*, encoding an improved mutant tyrosinase. To direct the carbon flow from central metabolism into the common aromatic and the L-tyrosine biosynthetic pathways, feedback inhibition resistant versions of key enzymes were expressed in strains lacking the sugar phosphotransferase system and TyrR repressor. The expressed tyrosinase consumed intracellular L-tyrosine, thus causing growth impairment in the engineered strains. To avoid this issue, a two phase production process was devised, where tyrosinase activity was controlled by the delayed addition of the cofactor Cu. Following this procedure, 3.22 g/L of melanin were produced in 120 h with glucose as carbon source. Analysis of produced melanin by Fourier transform infrared spectroscopy revealed similar characteristics to a pure eumelanin standard.

**Conclusions:**

This is the first report of a process for producing melanin from a simple carbon source at grams level, having the potential for reducing production cost when compared to technologies employing L-tyrosine as raw material.

## Background

Melanins constitute a diverse class of aromatic polymers synthesized by organisms from most biological groups [1]. The biological functions of this class of compounds are various and mainly related to protection from environmental stresses [2]. Eumelanin, a common type of melanin, is synthesized from L- tyrosine by copper containing enzymes called tyrosinases (monophenol monooxygenase EC 1.14.18.1). Using molecular oxygen, these enzymes catalyze the hydroxylation of L-tyrosine to L-dihydroxyphenylalanine (cresolase activity) and its subsequent oxidation to dopachrome (catecholase activity). Dopachrome non-enzymatically oxidizes and polymerizes to form eumelanin [3].

The characterization of melanins has revealed properties having potential industrial applications. Melanins can act as UV absorbers, amorphous semiconductors, cation exchangers, X-ray and γ-ray absorbers [2,4]. Therefore, there is considerable interest in the development of biotechnological processes for the production of these polymers. Production of melanins has been achieved in liquid cultures of naturally melanogenic and also with recombinant microorganisms [5-7]. The expression in *Escherichia coli* of heterologous genes encoding tyrosinases from *Streptomyces antibioticus* or *Rhizobium etli* has resulted in the generation of strains capable of transforming L-tyrosine or other substrates into melanins [7-9]. Production processes based on these recombinant microorganisms consist in the bioconversion of supplemented L-tyrosine into melanin in cultures with minimal or complex media.

Metabolic engineering of *E. coli* and other bacterial species has been applied to the generation of strains that overproduce L-tyrosine, employing simple sugars as raw material [10,11]. These strains have the potential for total synthesis of melanin if they also expressed a tyrosinase activity. Recently, a method was developed to enable correlation of melanin production with the amount of L-tyrosine synthesized by engineered strains that express a tyrosinase from *R. etli*, thus demonstrating the feasibility of melanin production from glucose in *E. coli*[12]. However, since this method was developed for screening purposes, melanin formation from glucose was only verified by visual inspection in Petri dishes.

**Figure 1:**
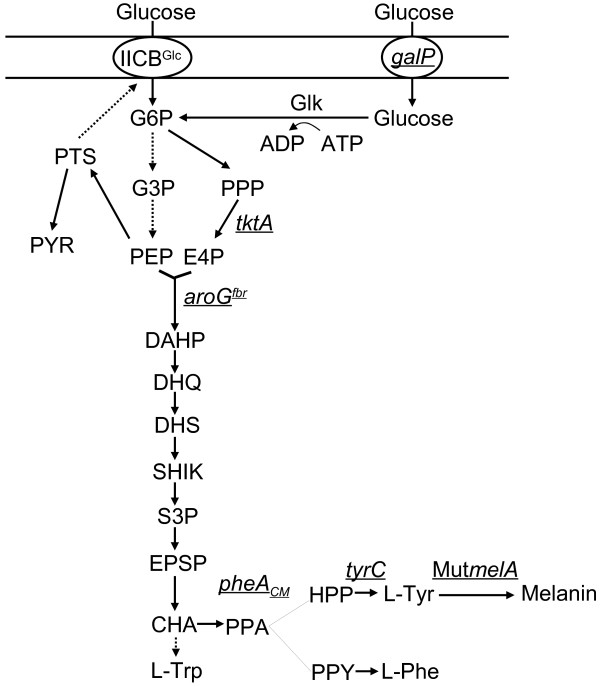
**Metabolic pathways related to tyrosine and melanin biosynthesis in recombinant *Escherichia coli*.** Dashed arrows indicate two or more enzyme reactions. Underlined genes were overexpressed from plasmids (*aroG*^*fbr*^, *tktA*, *pheA*_*CM*_, *tyrC* and Mut*melA*) or the chromosome (*galP*). Abbreviations: PTS, enzyme I and phosphohistidine carrier protein; IICB^Glc^, integral membrane glucose permease; *galP*, galactose permease; Glk, glucokinase; *tktA*, transketolase; ATP, adenosine triphosphate; ADP, adenosine diphosphate; G3P, glyceraldehyde-3-phosphate; G6P, glucose-6-phosphate; E4P, D-erythrose 4-phosphate; PEP, phosphoenolpyruvate; PYR, pyruvate; DAHP, 3-deoxy-D-*arabino*-heptulosonate 7-phosphate; DHQ, 3-dehydroquinic acid; DHS, dehydroshikimate; SHIK, shikimate; S3P, shikimate 3-phosphate; EPSP, 5-enolpyruvylshikimate-3-phosphate; CHA, chorismate; PPA, prephenate; HPP, 4-hydroxyphenylpyruvate; PPY, phenylpyruvate; L-Tyr L-tyrosine; L-Phe L-phenylalanine; L-Trp, L-tryptophan; *aroG*^*fbr*^, feedback inhibition resistant DAHP synthase; *tyrC*, cyclohexadienyl dehydrogenase; *pheA*_*CM*_, chorismate mutase domain from chorismate mutase-prephenate dehydratase.

An strategy for generating a melanin production strain can be divided in two steps: First, metabolic engineering must be applied to increase carbon flow into the L-tyrosine biosynthetic pathway. A second step involves the expression of a gene encoding a tyrosinase to a level sufficient for transforming L-tyrosine into melanin. The general strategy for generating L-tyrosine microbial production strains involves genetic modifications to redirect carbon flow from central metabolism into the common aromatic pathway by expressing a feedback insensitive version of the enzyme 3-deoxy-D-*arabino*-heptulosonate 7-phosphate (DAHP) synthase (Figure 1). Redirection of carbon flow from the common aromatic pathway into the L-tyrosine biosynthetic pathway can be accomplished by expressing genes encoding feedback insensitive versions of enzymes chorismate mutase (CM) and prephenate dehydrogenase (PDH) [10,11]. Additional modifications aimed at strain performance improvements include increasing the availability of the precursors phosphoenolpyruvate (PEP) and D-erythrose 4-phosphate (E4P), that are condensed by DAHP synthase in the first step of the common aromatic pathway (Figure 1). It is known that PEP consumption by the PEP:sugar phosphotransferase system (PTS) during glucose import limits the yield in the synthesis of aromatic compounds in *E. coli*[13,14]. Therefore, with the aim of avoiding PEP consumption during glucose import, *E. coli* mutant strains lacking PTS activity have been generated where glucose is internalized by the galactose permease (GalP) and phosphorylated by glucokinase (Glk) using ATP as the phosphate donor [15]. These mutant *E. coli* strains, having a PTS^-^ glucose^+^ phenotype, display higher aromatics yield from glucose when compared to a wild type PTS^+^ strain [16]. Further improvement of L-tyrosine *E. coli* production strains has been achieved by deleting the gene coding for TyrR. This protein is a transcriptional regulator that represses transcription of several genes encoding enzymes that participate in L-tyrosine synthesis [11,17]. TyrR inactivation in a strain modified for L-tyrosine production increased 1.7-fold the specific rate of production for this amino acid [18].

An *E. coli* strain that produces L-tyrosine from a simple carbon source can be transformed to a melanin producer by expressing a gene coding for a tyrosinase. In principle, this strain should enable the development of a biotechnological process for melanin production. However, as previously reported, the generation of a production strain and a fermentation process for melanin production involves several specific challenges. As shown by several groups, melanin production occurs only when cells enter the stationary phase [8,9,12]. It is not yet known what factors limit melanin production in the earlier growth phases. It has also been determined that tyrosinase from *R. etli* is rapidly inactivated at 37°C, therefore, melanin production cultivations must be performed at 30°C, a temperature not optimal for fast growth of *E. coli*. Finally, the processes involved in melanin formation during its production in a recombinant bacterium are still not known. It is not clear how the melanin precursors exit the cell and whether they polymerize in the periplasm, the external medium or both compartments. Furthermore, melanin precursors are reactive species that could form covalent bonds with other cellular metabolites; therefore, the resulting aromatic polymer could display a chemical composition different from melanin derived from pure L-tyrosine.

In the present work, metabolic engineering and fermentative process strategies were applied to overcome some of the challenges involved in the production of an aromatic polymer similar to melanin in *E. coli*. This work was based on developing *E. coli* strains engineered for L-tyrosine production as a platform to express a gene encoding an improved mutant version of a tyrosinase from *R. etli.* By employing a two-phase production strategy, a final melanin titer of 3.22 g/L was generated in bioreactor cultures with glucose as carbon source.

## Methods

### Bacterial strains and plasmids

The *E. coli* strains and plasmids employed in this study are described in Table 1. W3110 is an *E. coli* wild type strain, employed in this study as a parental host [19]. VH33 is a PTS^-^ glucose^+^ strain derived from W3110, which recovered the ability to grow on glucose as a result of replacing the native promoter region of *galP* in the chromosome by the strong *trc* promoter [20]. The GalP symporter protein together with glucokinase activity can replace PTS function in a PTS^-^ strain. Strain VH33*tyrR* was obtained by deleting the *tyrR* gene in VH33 [18]. Plasmid pRW300 carries the *aroG*^fbr^ gene encoding a feedback inhibition resistant version of the enzyme DAHP synthase [16]. pCL*tktA* contains the *tktA* gene which encodes transketolase A, involved in E4P biosynthesis [16]. Plasmid pTrc*tyrCpheA*_CM_ carries gene *tyrC* encoding the feedback inhibition-insensitive enzyme cyclohexadienyl dehydrogenase (TyrC) from *Zymomonas mobilis* and gene *pheA*_*CM*_ encoding the chorismate mutase domain from chorismate mutase-prephenate dehydratase (PheA_CM_) from *E. coli*[10]. These genes form an operon that is transcribed from the *trc* promoter.

**Table 1:** ***E. coli *** **strains and plasmids used in this study**

**Strain or plasmid**	**Relevant features**	**Reference or source**
W3110	F^-^, λ^-^, INV (*rnnD-rnnE*)1	ATCC27325
VH33	W3110 Δ*ptsH, ptsI, crr*::km, Δ*lacI, lacZ*::*lox*P, Ptrc-*galP*	[20]
VH33*tyrR*	VH33 Δ*tyrR*::FRT	[18]
W3110M	W3110/pACMut*melA*I	This work
W3110MG	W3110/pRW300, pACMut*melA*I	This work
W3110MGT	W3110/pRW300, p*MmelAtyrCpheA*_*CM*_	This work
VH33MGT	VH33/ pRW300, p*MmelAtyrCpheA*_*CM*_	This work
VH33GTR	W3110tyrR/pRW300, pACYC*tyrCpheA*_*CM*_	This work
VH33MGTR	VH33tyrR/pRW300, p*MmelAtyrCpheA*_*CM*_	This work
VH33MGTRK	VH33tyrR/pRW300, pACYC*tyrCpheA*_*CM,*_ pCL*tktA*	This work
Plasmids	Relevant features	Reference or source
pTrc99A	Expression plasmid carrying the *trc* promoter, a multiple cloning site, the T1 and T2 *rrnB* terminator sequences, the *bla* and *lacI*^q^ genes.	[21]
pACYC184	Cloning plasmid carrying genes *cat* and *tet*. p15A replication origin.	[22]
pRW300	*aroG*^*fbr*^ under control of the *lacUV5* promoter carries *lacI*^q^ and *tet* genes. Replication origin from pBR322.	[16]
pCL*tktA*	Carried genes *tktA* and *aada*. Derivative of plasmid pCL920 with pSC101 origin of replication.	[16]
pTrcMut*melA*	Gene Mut*melA* cloned in pTrc99A.	[9]
pTrc*tyrCpheA*_*CM*_	*tyrC* and *pheA*_*CM*_ under the control of *trc* promoter. *pheA*_*CM*_ codes for the chorismate mutase domain of P-protein.	[10]
pACMut*melA*I	Gen Mut*melA* cloned in pACY184.	This work
pMut*melAtyrCpheA*_*CM*_	Genes *tyrC* and *pheA*_*CM*_ cloned in pACMut*melAI*.	This work
pACYC*tyrCpheA*_*CM*_	Genes *tyrC* and *pheA*_*CM*_ cloned in pACYC184.	This work

### Construction of pACMutmelAI, pMutmelAtyrCpheA_CM_ and pACYCtyrCpheA_CM_

The *melA* gene encodes for a tyrosinase from *Rhizobium etli* CFN42 [8]. MutMelA is a modified version of MelA tyrosinase, having a single nucleotide change that resulted in the replacement of Asp_535_ by Gly. This mutant was selected during *melA* cloning. In plates and liquid bioreactor cultures, the onset of melanin production occurs earlier in strains expressing Mut*melA* when compared to a strain expressing wild-type *melA*. However, the rate of melanin synthesis is the same with either version of the tyrosinase [9]. Plasmid pACMut*melA*I was constructed by ligating a 2.5 kb *Ssp*I fragment from pTrcMut*melA* to plasmid pACYC184 digested with *Eco*RV. The 2.5 kb fragment contained the *trc* promoter, Mut*melA* gene and the three transcriptional terminators from pTrc99A [21]. In order to subclone *tyrC* and *pheA*_*CM*_ genes into pACMut*melA*I, plasmid pTrc*tyrCpheA*_*CM*_ was digested with *Ssp*I to generate fragments of 3524 and 1916 bp. The 1916 bp fragment contained the *trc* promoter, *tyrC*, *pheA*_*CM*_ genes, and the three transcriptional terminators from pTrc99A. Subsequently, plasmid pACMut*melA*I was digested with *Bstz*17I and ligated to pTrc*tyrCpheA*_*CM*_ digested with *Ssp I* to generate plasmid pMut*melAtyrCpheA*_*CM*_. Finally, in order to eliminate the Mut*melA* gene, plasmid pMut*melAtyrCpheA*_*CM*_ was digested with B*amH* I. The B*amH* I digestion generated two fragments: the fragment of 2120 bp, corresponded to the expected size for the Mut*melA* gene. The second fragment of 6506 bp contained *tyrC* and *pheA*_*CM*_ genes. This fragment was gel purified and self-ligated to generate pACYC*tyrCpheA*_*CM*_.

### Cultivation media and growth conditions for cultures for L-tyrosine and melanin production with recombinant *E. coli* strains

Selection of transformant cells was performed in Luria-Bertani (LB) medium. Antibiotics (Ab) were used at the following concentrations, per ml: ampicillin (Ap), 200 μg for pTrc99a derivatives. Tetracycline (Tc), 30 μg for pRW300; and chloramphenicol (Cm), 30 μg for pACYC184 derivatives.

**Figure 2:**
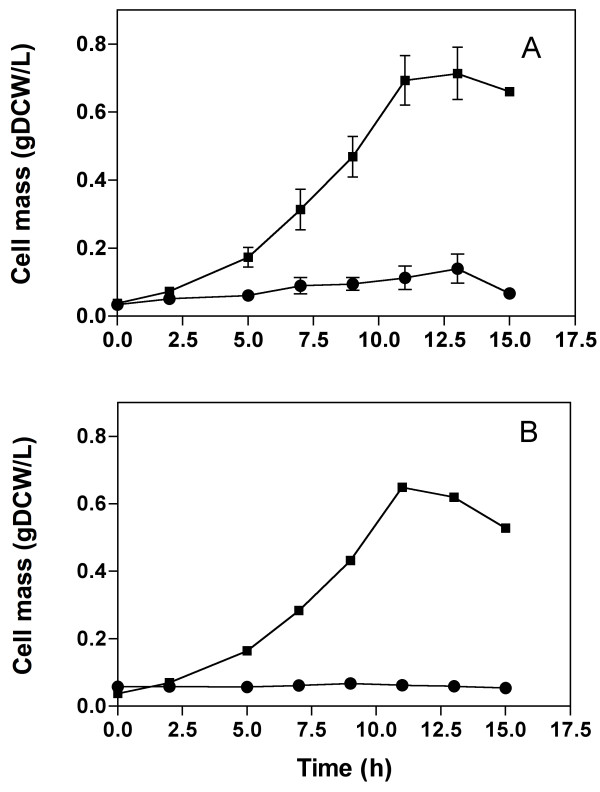
**Growth kinetics in minimal medium, 2 g/L of glucose. CuSO**_**4 **_**was added at the beginning of the culture.** Experiments were carried out in shaken flasks with L-Tyrosine or (■) without L-Tyrosine (●). **(A)** W3110M and **(B)** W3110MG. Graphs represent the mean of two independent experiments.

**Figure 3:**
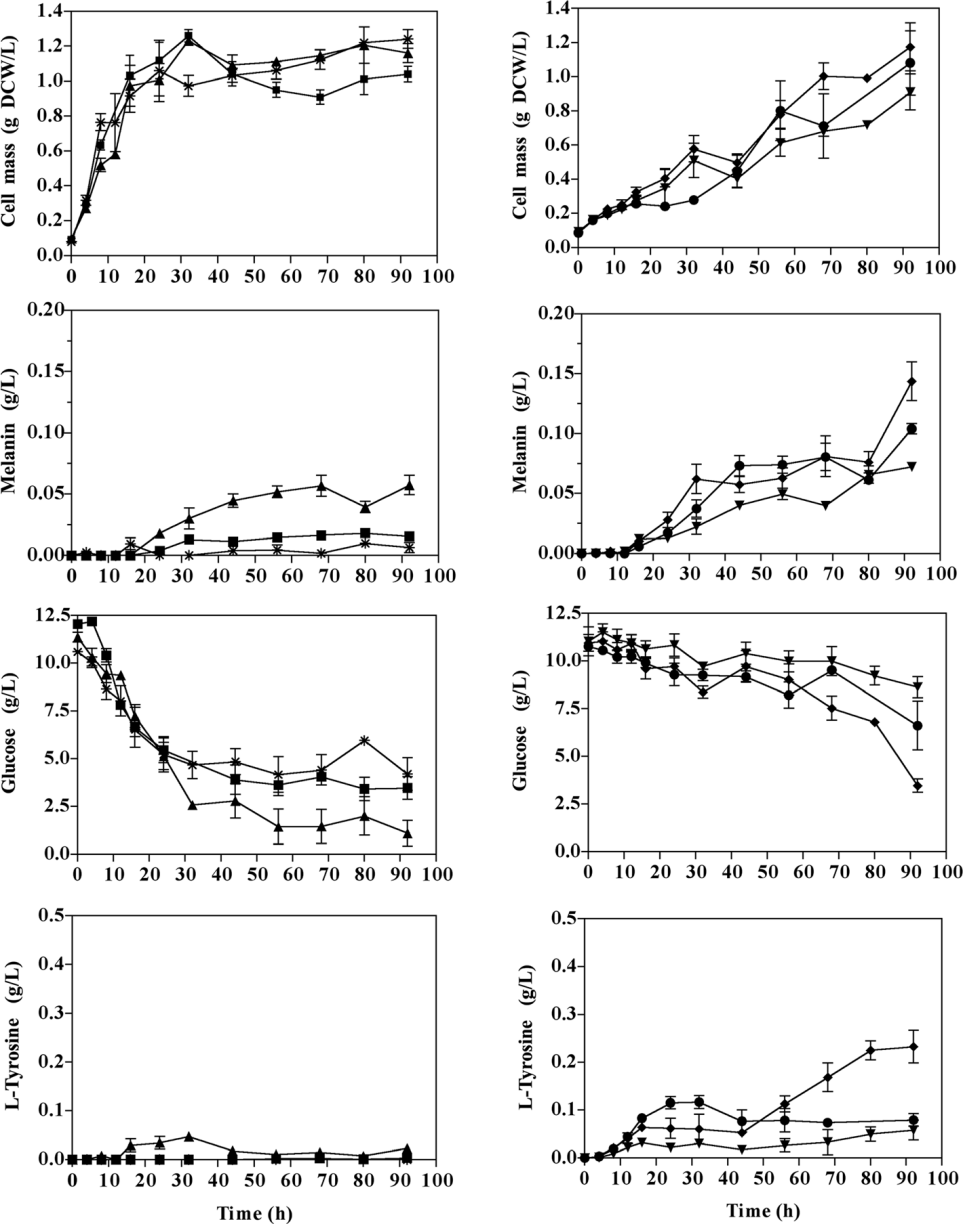
**Culture profiles carried out in shaken flasks with strains modified for melanin production from glucose (ca. 10 g/L). CuSO**_**4 **_**was added at the beginning of the stationary phase.** Symbols for strains: *W3110M, ■ W3110MG, ▲ W3110MGT, ▼VH33MGT, ♦ VH33MGTR and ● VH33MGTRK. Graphs represent the mean of three independent experiments.

**Figure 4:**
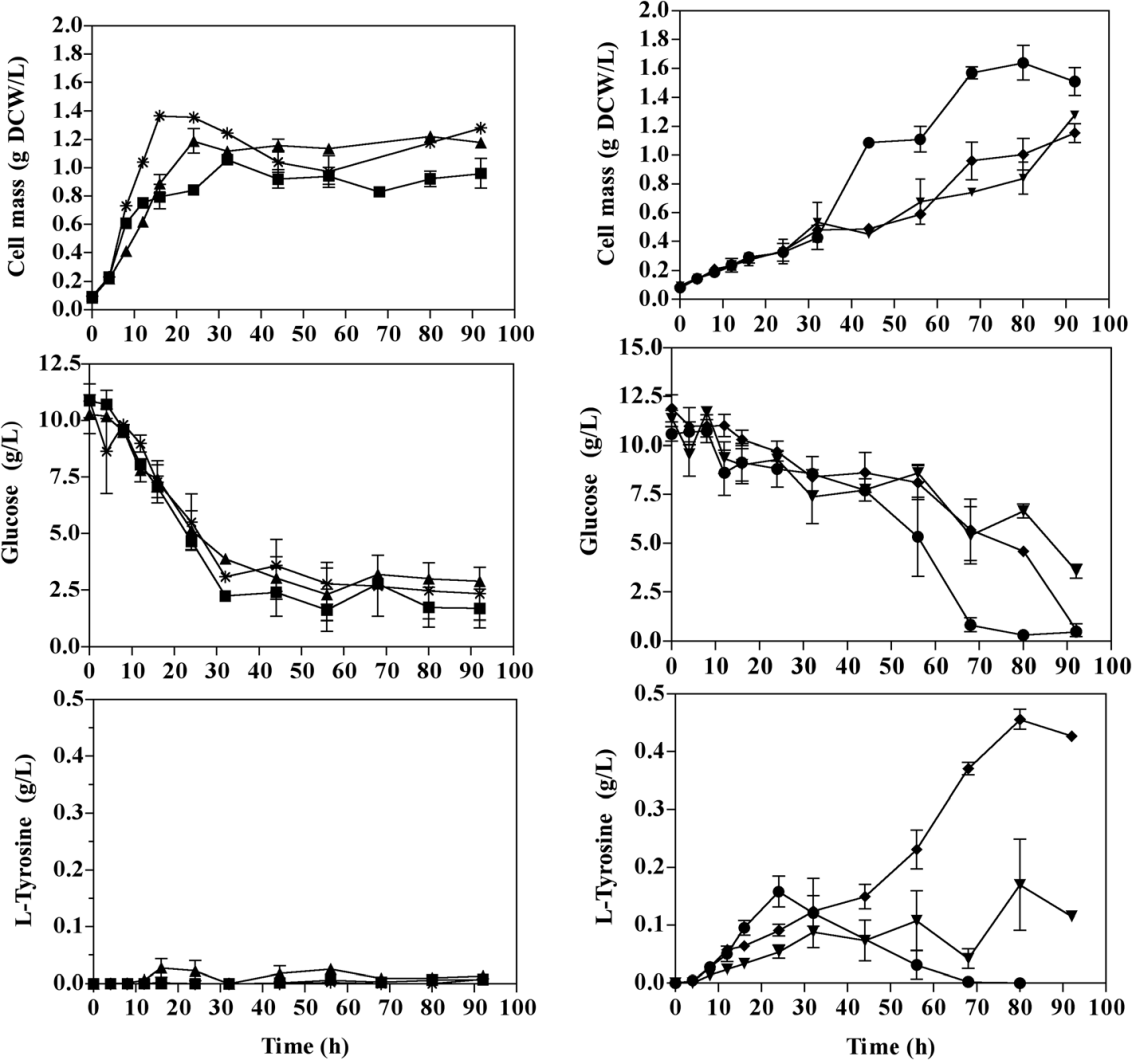
**Culture profiles carried out in shaken flasks with strains modified for L-tyrosine production from glucose (ca. 10 g/L) in medium lacking CuSO**_**4 **_**(tyrosinase is inactive without CuSO**_**4**_**).** Symbols for strains: *W3110M, ■ W3110MG, ▲ W3110MGT, ▼VH33MGT, ♦ VH33MGTR and ● VH33MGTRK. Graphs represent the mean of two independent experiments.

Cultures for the production of melanin from L-tyrosine were performed at 30°C and 300 rpm agitation in 250 mL baffled shake flasks with 50 mL of M9 minimal salts medium [23] supplemented with 2 or 10 g/L of glucose, 0.1 mM IPTG, the required antibiotic for each strain and 0.4 g/L of L-tyrosine. 20 μg/mL CuSO_4_ were added at the beginning or at 16 h of culture time, as indicated in Figures 2 and 3. Kinetics for the production of melanin from glucose were carried out under the previously described conditions, but L-tyrosine was not added to culture media (Figure 3). Culture conditions for the production of L-tyrosine were the same as those employed for melanin production from glucose, except that CuSO_4_ was not added to the shake flasks culture medium (Figure 4). Bioreactor cultures for melanin production were started at an optical density at 600 nm of 1.0 from an inoculum grown overnight in M9 medium supplemented with 1% tryptone, 2 g/L of glucose and the required antibiotics for each strain. Cultures were performed using a working volume of 500 ml in 1-L stirred tank bioreactors model ADI 1010 (Applikon, The Netherlands). Culture medium employed was M9 supplemented with three pulses of glucose each one of 20 g/L. Air flow rate was maintained at 1 vvm and O_2_ partial pressure above 40%. Dissolved oxygen was measured with a polarographic oxygen probe (AppliSens; Applikon, Inc., Foster City, CA, USA). Based on previous studies, initial culture temperature was maintained at 32°C and then reduced to 30°C at the point when culture was in mid-exponential phase, corresponding to a biomass concentration between 5 and 6 g/L [9]. During these cultures, the pH was maintained at 7.0 and increased to 7.5 at mid-exponential phase. At this point, CuSO_4_ was added to a final concentration of 20 μg/mL.

### Analytical methods

Eumelanin shows an absorbance peak at 600 nm, therefore, cell growth was monitored by subtracting the absorbance at 600 nm of supernatants to the 600 nm absorbance of the non-centrifuged cell culture sample [9]. Dry cell weight was calculated multiplying the 600 nm absorbance by a previously determined coefficient factor of 0.37 g/L [9]. Melanin production was determined by measuring absorbance at 400 nm from culture supernatants. One OD_400_ unit is equivalent to 0.066 g/L of eumelanin [9]. Glucose, glycerol, 3-dehydroshikimate (DHS) and shikimate (SHIK) concentrations were determined by using high performance liquid chromatography with an Aminex HPX-87H column (300 × 7.8 mm; 9 mm) (Bio Rad, CA, USA) in a HPLC system: 600E quaternary bomb, 717 automatic injector, a refraction index detector and a 996 photodiode array detector (Waters Co., USA). Running conditions were: mobile phase, 5 mM H_2_SO_4_; flow, 0.5 ml/min and temperature, 50°C. Glucose was identified by refraction index. L-tyrosine concentration was measured using a Phenomenex Synergy Hydro RP18 column (150 × 4.6 mm, 4 μm) (Sigma, St. Louis MO, USA) in an Agilent HPLC system series 1100 (Agilent Technologies, Palo Alto, Ca). Running conditions were: mobile phase, 0.1% acetic acid in 10% methanol; flow, 0.5 ml/min and temperature, 50°C. L-Tyrosine was measured using an UV detector at 280 nm. It is important to point out that L-tyrosine is insoluble in water at concentrations above 0.4 g/L at pH 7.0. Therefore, to solubilise this amino acid, 50 μl of HCl 6 N per ml of culture broth were added to the samples. Subsequently, samples were mixed and incubated at 42° and centrifuged for 30 min. Finally, to eliminate bacterial cells, samples were centrifuged for 5 min at 12000 rpm and the supernatant was employed for determining L-tyrosine concentration. Detection and quantification of L-phenylalanine, L-tryptophan, L-tyrosine and chorismate (CHO) was performed using a Supelco Discovery C18 column (250 × 4.6 mm, 5 μm) (Sigma, St. Louis MO, USA) in an Agilent HPLC system series 1100 that was linked to a mass spectrometer (Agilent LC/MSD series 1100). Running conditions were: mobile phase, 1% acetic acid in 40% methanol; flow, 0.5 ml/min. The effluent was introduced in to the Es source with the following conditions: the capillary voltage was set at 3000 V, a flow of heated nitrogen gas of 200°C was maintained at 13 L/min. Melanin samples employed for Fourier transform infrared spectroscopy (FTIR) analyses were obtained from cultures of strains VH33MGTR and VH33MGTRK. These strains were growth in shake flasks with 1 L of M9 minimal salts medium, supplemented with a mixture of 5 g/L of glucose and 5 g/L of glycerol, 0.1 mM IPTG, at 30°C and 300 rpm. CuSO_4_ was added at 16 h of culture. After 48 h of culture time; cells were separated from supernatants by centrifugation at 4500 r.p.m, for 5 min at 4°C. The melanin was precipitated from the supernatant by adjusting pH to 2.0 with HCl 1 N and centrifuging at 12000 rpm for 10 min and 20°C. The precipitate was rinsed with distilled water and then dried at 42°C for 48 h. FTIR spectra were performed at room temperature using a Nicolet Magna 750 spectrometer with 4 cm^-1^ of resolution and 100 scans per spectrum. The samples were finely ground and dispersed in KBr with a ratio of 1:100, and then they were pressed as a wafer of ~25 mg/cm^2^. Samples were designated as D for melanin produced by VH33MGTR, and E for the melanin synthesized by VH33MGTRK. A commercial eumelanin sample from Sigma was used as reference material.

## Results and discussion

### Effect of expressing *MutmelA* and *aroG*^*fbr*^ on melanin production and growth capacity in strains W3110M and W3110MG

To determine the phenotypes leading to a high capacity for melanin production from glucose, a series of strains were generated that expressed the *melA* gene from *R. etli*, and also with modifications known to have a positive impact on L-tyrosine production. The first step in strain characterization consisted on determining the capacity of strain W3110M (carrying plasmid pACMut*melA*I) to transform L-tyrosine into melanin in shake flask cultures. This experiment would provide a reference value for the maximum rate of melanin production resulting from Mut*melA* expression in this strain when L-tyrosine is non-limiting. W3110M produced melanin from L-tyrosine at a specific rate of production (q_Mel_) of 36.6 mg/g_DCW_/h with a yield from L-tyrosine (Y_Mel/L-Tyr_) of 1.07 g/g, corresponding to the complete conversion of L-tyrosine to melanin. The first evaluation of strains W3110M and W3110MG for producing melanin from glucose was performed in shake flask cultures employing M9 minimal salts medium supplemented with glucose 2 g/L, IPTG and CuSO_4_. To determine the maximum melanin production capacity in these strains, cultures were also performed in the same medium but with added L-tyrosine 0.4 g/L. In medium containing supplemented L-tyrosine, both strains grew well and produced melanin, as expected (Figure 2). However, a 66% reduction in the specific growth rate (μ) for strain W3110M and a lack of growth of strain W3110MG was observed in the medium lacking L-tyrosine. These results indicate that tyrosinase activity has a negative effect on growth capacity in these two strains. Since growth impairment can be alleviated by the presence of L-tyrosine, it can be assumed that tyrosinase activity is consuming intracellular L-tyrosine, thus causing a phenotype resembling a partial or total auxotrophy for this amino acid in these two strains. It was further determined that all other *E. coli* W3110 derivative strains employed in this study (W3110MGT, VH33MGT, VH33MGTR, VH33MGTRK) did not show growth in M9 medium lacking L-tyrosine (data not shown). The negative effect on growth rate can be explained by the combined result of L-tyrosine consumption by tyrosinase and the metabolic burden caused by the over expression of genes involved in L-tyrosine and melanin production. In these strains, carbon flow to the common aromatic pathway is enhanced, which should lead to a higher L-tyrosine level when compared to strain W3110M. To determine the extent of carbon flow increase into the common aromatic pathway resulting from expression of *aroG*^fbr^, cultures were performed with strains W3110MG and W3110M, and samples from supernatants were subjected to HPLC analysis. Since previous data showed that tyrosinase activity has a negative effect on growth capacity in this strain, cultures were grown using minimal medium lacking CuSO_4_. Previous reports had shown that absence of the tyrosinase cofactor Cu in culture medium, results in the synthesis of inactive tyrosinase [8]. Therefore, even if gene Mut*melA* is being expressed and tyrosinase protein is synthesized, in the absence of Cu the enzyme is not active. Under these culture conditions, it was found that growth of strain W3110MG was not impaired in minimal medium lacking L-tyrosine. This result confirmed that tyrosinase activity was causing depletion of intracellular L-tyrosine. When analyzing supernatants from cultures of strain W3110M, final products of the aromatic pathways were not detected. This is an expected result, since wild-type *E. coli* does not secrete either of these compounds. In contrast, at 16 h of cultivation, in the supernatant of cultures with strain W3110MG the specific concentrations of DHS, SHIK, CHO, L-Phe, L-Tyr and L-Trp were 13.8, 18.4, 54, 21.7, 4.5 and 0.5 mg/g DCW, respectively. These results show, as expected, that expression of *aroG*^fbr^ increased carbon flow into the common aromatic pathway, resulting in the accumulation and secretion of intermediates and aromatic amino acids. These data also indicate that the observed increase in L-tyrosine synthesis in strain W3110MG is not sufficient for overcoming its depletion by tyrosinase, therefore, growth impairment was observed.

### Characterization of strains expressing genes involved in L-Tyr and melanin synthesis

As it was shown above, if Cu is not added to the culture medium, tyrosinase enzyme is synthesized but it is not active. Considering this information, a two-stage culture strategy was devised for melanin production in minimal salts medium: During the first stage of the culture, the medium would lack Cu, thereby allowing growth of the recombinant strains. Once the culture entered the late-exponential phase, then CuSO_4_ would be added to the medium to activate the already-synthesized tyrosinase and to allow synthesis *de novo* of active tyrosinase. Figure 3 shows the results of shake-flask experiments performed following this strategy with strains W3110M, W3110MG, W3110MGT, VH33MGT, VH33MGTR and VH33MGTRK. As it can be seen in Table 2, strain W3110M did not produce melanin. This result indicates that tyrosinase expression alone is not sufficient to cause synthesis from glucose of a detectable amount of melanin in *E. coli* W3110. In contrast, 15.1 mg/L of melanin were produced from glucose in cultures with strain W3110MG. This positive effect on melanin accumulation is the result of expressing gene *aroG*^*fbr*^, thus alleviating feedback inhibition at the first enzymatic step of the common aromatic pathway and causing an increase in carbon flow from central metabolism into aromatics (Figure 1). Strain W3110MGT expresses *aroG*^*fbr*^, *tyrC* and *pheA*_*CM*_ encoding enzymes that increase carbon flow to the common aromatic and L-tyrosine biosynthetic pathways. This strain produced 2.6-fold more melanin when compared to W3110MG. As expected, the expression of feedback-insensitive versions of enzymes that direct carbon flow to the L-tyrosine pathway caused a clear increase in melanin production. Strain VH33MGT, a PTS^-^ glucose^+^ derivative of W3110 that expresses *aroG*^*fbr*^, *tyrC* and *pheA*_*CM*_, produced 1.8-fold more melanin than W3110MGT. This result corroborates the positive effect in *E. coli* of PTS inactivation on aromatics production when employing glucose as carbon source, as previously observed [14]. In the PTS^-^ glucose^+^ background, inactivation of *tyrR* (strain VH33MGTR) caused a further 2.1 fold increase in melanin production when compared to VH33MGT, thus showing the positive effect of eliminating TyrR repression on several genes of the common aromatic and L-tyrosine pathways. Strain VH33MGTRK bears the previously mentioned modifications and in addition, the gene encoding transketolase was overexpressed. This modification did not cause an increase in the level of produced melanin. This is an unexpected result, since *tktA* overexpression has been shown to increase aromatics production capacity by avoiding limitation in E4P availability [13,24]. A possible explanation for this result could be that E4P is non-limiting in these strains under the studied conditions. Alternatively, a metabolic burden effect caused by the over expression of five genes in this strain could negate the potential positive effect of increased *tktA* expression.

**Table 2:** Melanin and L-tyrosine production in shake flask cultures with mineral medium supplemented with 10 g/L glucose*

**Strain**	**Melanin titer**	**L-TYR**	**L-TYR max**^**1**^	**L-Tyr/melanin conversion**
	**(mg/L)**	**(mg/L)**	**(mg/L)**	**(%)**
W3110M	0	0	0	0
W3110MG	15.1 ± 3.7	7.10 ± 4.5	6.9 ± 6.4	198
W3110MGT	39.9 ± 2.6	27.5 ± 12.1	55.1 ± 14.9	66
VH33MGT	72.4 ± 1.5	70.6 ± 13.9	91.0 ± 2.5	72
VH33MGTR	155 ± 34.8	263 ± 38.9	456 ± 29.4	30
VH33MGTRK	112 ± 14.8	66.4 ± 0.93	158 ± 46.3	64

*CuSO_4_ was added to culture medium 16 h after inoculation.

^1^Cultures were performed in medium lacking CuSO_4_.

To determine the maximum capacity for L-tyrosine synthesis from glucose by each of the characterized strains, experiments were performed under the same previous conditions but differing in the lack of addition of CuSO_4_ to the culture medium. As shown in Table 2 and Figure 4, L-tyrosine was found in culture supernatants of all strains, with the exception of W3110M, as expected. The amount of L-tyrosine showed a positive correlation with the melanin titer produced in the conditions where tyrosinase was active. By performing cultures in the presence or absence of CuSO_4_ a conversion yield of L-tyrosine to melanin could be estimated. As shown in Table 2, the yield value widely differed among strains, with a tendency towards lower conversion efficiency in strains with the higher melanin titers. With the exception of strain W3110MG, all other strains displayed a higher L-tyrosine than melanin production capacity. The accumulation of L-tyrosine in cultures where tyrosinase is active is an indication of a metabolic bottleneck where the rate of synthesis of this amino acid surpasses its rate of consumption by tyrosinase. This is evident in nearly all characterized strains (Figure 3). Strain VH33MGTR showed the highest levels of melanin and L-tyrosine accumulation and therefore, this means a higher potential for melanin production improvement by increasing tyrosinase activity or by means of cultivation process optimization aimed at coupling L-tyrosine and melanin formation processes.

### Characterization of the produced melanin

**Figure 5:**
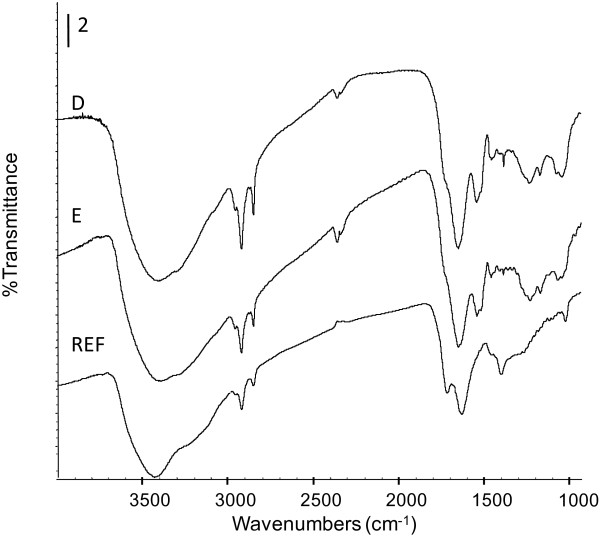
**Fourier transform infrared spectroscopy spectra of control melanin and samples produced by engineered *E. coli *strain: D, melanin produced by W3110MGTR. E, melanin produced by W3110MGTRK. REF, eumelanin standard.** Melanin produced by engineered *E. coli* strains were purified as described in the Materials and Methods section.

FTIR spectroscopy experiments were performed to obtain information about the chemical structure of the produced melanin. Melanin samples where obtained from shake flask cultures with strains VH33MGTR (sample D) and VH33MGTRK (sample E). Figure 5 shows the IR spectra for different samples. In the high frequency region (3200–3600 cm^-1^), all samples show a broad absorption band assigned to O-H y N-H stretching vibrations. The high intensity and broadening of this band is assigned to hydrogen bonding between hydroxyl and amine groups. These hydroxyl groups could be bonded to C (C-OH bond) due to the presence of IR bands in the 1000–1114 cm^-1^ region.

In the ν_(C-H)_ mode (2800–3000 cm^-1^), absorptions bands at 2955 (vw), 2919(s) and 2850 (m) cm^-1^ are observed. These bands are associated to ν_as(C-H3)_, ν_as(C-H2)_ and ν_s(C-H2)_ respectively which are typical of aliphatic organic compounds and their assignation is corroborated by the presence of the bands at 1455 and 1380 cm^-1^ corresponding to δ_as(C-H)_ and δ_s(C-H)_ vibrations modes. It is evident the changing in the bands intensity from one sample to another showing higher IR absorption for D and E samples. These bands and the ones present in the 1050–1250 cm^-1^ region could be due to some metabolites produced by the *E. coli* strains employed in this study (i.e. chorismate and L-phenylalanine) [10].

The analysis of the 1828–1480 cm^-1^ zone shows two well defined maxima at 1715 and 1624 cm^-1^ for the reference sample. These two maxima were assigned to carbonyl stretching (C = O) and to aromatic rings, C = C double bonds, conjugated with C = O and/or COO- groups respectively. For D and E samples, the band at 1715 cm^-1^ appears as a shoulder due to the displacement to higher frequencies of the band associated to C = C bonds (1624 cm^-1^) which now appears at about 1650 cm^-1^. This band also could be related to quinone compounds. Other bands localized from 1480 to 1325 cm^-1^ frequencies indicate that ionized carboxylic group (COO^-^) and free carboxylic groups are present in the melanin structures.

In summary, the FT-IR spectra of the produced melanin presented similar features to those of the standard sample. They all show the main absorbance peaks of functional groups, OH, NH, C = C, COOH and C = O of an eumelanin sample.

### Bioreactor cultures for melanin production with strains VH33GTR and VH33MGTR

**Figure 6:**
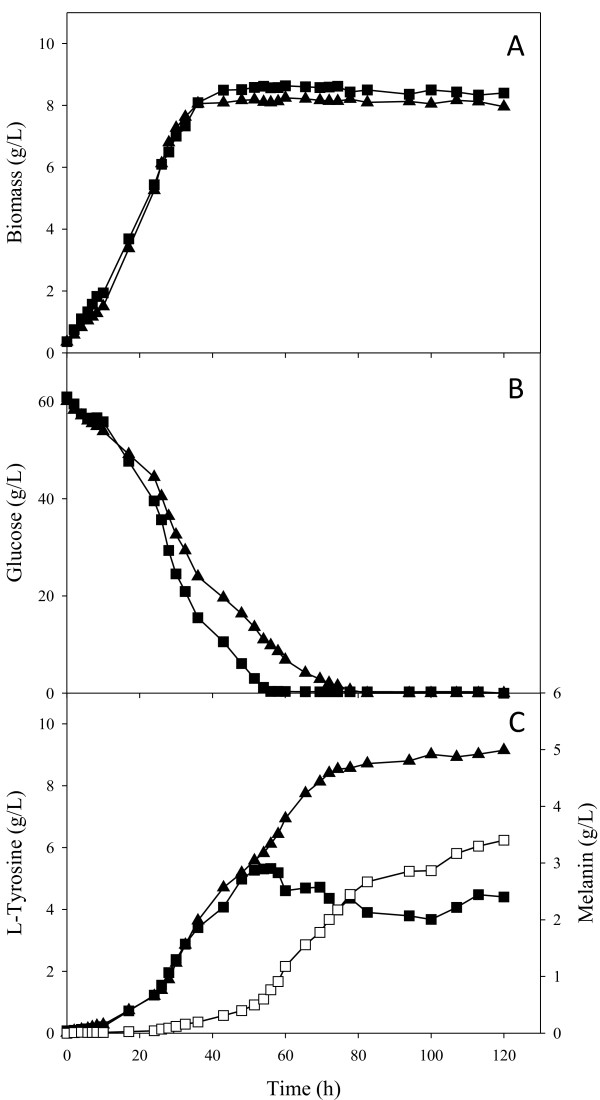
**Profiles from bioreactor cultures for the production of melanin and L-tyrosine using mineral media supplemented with glucose as carbon source. (A)** Biomass concentration, **(B)** glucose concentration, **(C)** L-tyrosine (filled symbols) and melanin (empty symbols) concentration. (▲) strain VH33GTR, (■) strain VH33MGTR. Graphs represent the mean of duplicate experiments. Percentage errors for data from strain VH33GTR were 4.48, 14.02 and 9.16% for biomass, glucose and L-tyrosine concentrations, respectively. Percentage errors for data from strain VH33MGTR were 15.64, 10.49, 8.03 and 16.07% for biomass, glucose, L-tyrosine and melanin concentrations, respectively.

Strains VH33MGTR and VH33GTR were cultured in a bioreactor to determine the effect of employing glucose as carbon source for melanin production. Strain VH33MGTR was chosen for these experiments since it displayed the best melanin production parameters in shake flask cultures. As a control, strain VH33GTR, also engineered for L-tyrosine production and lacking the gene Mut*melA*, was employed to determine maximum L-tyrosine production capacity under these culture conditions. A total amount of 60 g/L of glucose were supplemented to the minimal medium employed in these cultures. Under these conditions, a similar final cell mass concentration of approximately 8 g_DCW_/L was reached after 40 h of culture time, when the stationary phase started (Table 3, Figure 6). Specific rates of growth and substrate consumption were similar among both strains. Strain VH33GTR produced around 9 g/L of L-tyrosine after 120 h of cultivation. Under the same culture conditions, strain VH33MGTR displayed production of both L-tyrosine and melanin. This strain produced L-tyrosine until the 56^th^ h, and then accumulation stopped at 5.3 g/L. At this point, a relatively high rate of melanin accumulation was observed, coinciding with culture entry into the stationary phase. Melanin continued to accumulate until the end of the fermentation, reaching a final titer of 3.223 g/L. During the melanin production phase, a reduction of about 1 g/L of L-tyrosine in culture broth was observed from the 50^th^ until the 80^th^ h of culture time. This reduction is likely caused by L-tyrosine uptake and its conversion to melanin by the production strain; however, as shown by the control experiment, without Mut*melA* expression, it is likely that L-tyrosine was also produced from the 50^th^ until the 70^th^ h. The maximum theoretical yield of L-tyrosine from glucose is 0.533 g/g [11]. As a result of the incorporation of one oxygen atom per each L-tyrosine molecule by tyrosinase, a 15% increase in melanin mass is expected [9]. Therefore, the maximum theoretical yield of melanin from glucose is 0.636 g/g. In bioreactor cultures with VH33GTR the Y_Tyr/Substrate_ corresponded to 28% of the theoretical maximum. In the case of cultures with strain VH33MGTR, the Y_Mel/Substrate_ was equivalent to 14.6% of the theoretical maximum. However, if L-tyrosine plus melanin production are considered, then this value increases to 32.7%.

**Table 3:** Melanin and L-tyrosine production in bioreactor cultures with mineral medium supplemented with glucose as carbon source*

	**Final biomass concentration (g**_**DCW**_**/L)**	**μ**	**q**_**substrate**_	**q**_**Tyr**_	**q**_**Mel**_	**Y**_**Tyr/Substrate**_	**Y**_**Mel/Substrate**_	**Final L-tyrosine concentration**	**Final melanin concentration**
		**(h**^**-1**^**)**	**(g/g**_**DCW**_ **· h)**	**(g**_**Tyr**_**/g**_**DCW **_**h)**	**(g**_**Mel**_**/g**_**DCW**_ **· h)**	**(g**_**Tyr**_**/g**_**Sub**_**)**	**(g**_**Mel**_**/g**_**Sub**_**)**	**(g**_**Tyr**_**/L)**	**(g**_**Mel**_**/L)**
VH33GTR	8.077 ± 0.34	0.112 ± 0.00	0.130 ± 0.02	0.013 ± 0.00	N.D.	0.155 ± 0.00	N.D.	9.026 ± 0.16	N.D.
VH33MGTR	8.417 ± 0.05	0.129 ± 0.01	0.140 ± 0.00	0.013 ± 0.00	0.004 ± 0.00	0.100 ± 0.01	0.093 ± 0.01	4.152 ± 0.05	3.223 ± 0.35

*N.D., non detected.

**Figure 7:**
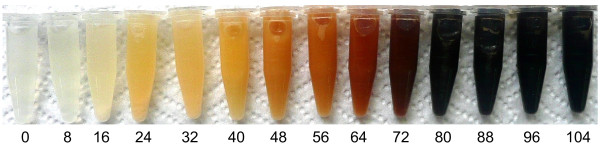
**1.5-mL tubes containing bioreactor culture samples at different elapsed times.** Number below each vial indicates sampling time in hours.

Figure 7 shows culture samples taken approximately every 8 h from previously described bioreactor experiments with strain VH33MGTR. As time progresses, a change of colour is evident. The last four samples show the expected black colour of melanin. The dynamics of melanin polymerization have not being determined in microbial production cultures. Nevertheless, it can be assumed that melanin polymer progressively increases in size as culture time proceeds and this is reflected in the colour of the pigment. These results indicate that it should be possible to obtain melanin at different stages of polymerization from these cultures. It is likely that melanin with different levels of polymerization will have distinct physicochemical properties, including range of spectrum absorption and water solubility [25].

In this study, an inexpensive carbon source was employed as raw material to determine strain performance parameters for melanin production. The detailed characterization of the generated production strains under different conditions has enabled the identification of potential targets for improvement. Most of the genetic modifications in these strains were chosen to increase carbon flow to the L-tyrosine pathway, leading to the accumulation of 9 g/L. However, the yield from glucose was a relatively low 28%. This result is likely caused by a metabolic bottleneck in the common aromatic pathway, as it has been reported previously [11]. Therefore, a strategy to increase strain performance should consider the identification and overexpression of genes coding for rate-limiting enzymes from this part of metabolism. The observed melanin yield from glucose in strain VH33MGTR was about half of that measured for L-tyrosine in strain VH33GTR. However, the yield resulting from adding L-tyrosine plus melanin in strain VH33MGTR showed a similar value to that observed for L-tyrosine in strain VH33GTR. It is also noteworthy that strain VH33MGTR accumulated in bioreactor cultures approximately 4 g/L of L-tyrosine in addition to 3.22 g/L of melanin. These results indicate that melanin production could be improved in VH33MGTR by increasing tyrosinase activity, thus avoiding L-tyrosine secretion and its extracellular accumulation. Tyrosinase enzymes are a diverse family that can be found in various biological groups. The search for tyrosinase enzymes with improved activity or higher temperature stability should be facilitated by employing a selection scheme based on melanin detection, as it has been reported for detecting improved L-tyrosine production strains [12].

## Conclusions

The various types of melanins are natural products having a wide range of potential applications as multifunctional polymers. The bioconversion of pure L-tyrosine or complex media components to melanin has been the basis for the biotechnological production of this type of polymer [7]. These efforts have resulted in the production of melanin at grams level, with the highest value reported as 6 g/L [9]. In the present study, up to 3.22 g/L of melanin were produced from glucose by employing an engineered strain and a production process based on delayed tyrosinase activation. These results constitute the basis for improvement regarding production cost over previous technologies, when considering the lower cost of glucose, when compared to L-tyrosine.

This work is the first example where engineered *E. coli* strains and a fermentative process resulted in the production, at grams scale, of an aromatic polymer from glucose with very similar chemical composition to a eumelanin standard. This production process should facilitate further research on the kinetics of melanin formation and the search for potential applications for this type of polymer.

## Competing interests

The authors declare that they have no competing interests.

## Authors’ contributions

MC, VB and GG participated in the design of this study. MC constructed the strains. MC and VB characterized the strains in flask and bioreactor cultures and analyzed the experimental data. AG participated in performing FTIR analysis of melanin samples. MC, VB, AG, AM, FB and GG participated in the analysis of the results as well as in writing and critical review of the manuscript. All authors have read and approved the manuscript.
